# Comparative Study on the Calcium Leaching Resistance of Low-Heat Cement, Moderate-Heat Cement, and Ordinary Portland Cement Pastes

**DOI:** 10.3390/ma18010212

**Published:** 2025-01-06

**Authors:** Chunmeng Jiang, Shihao An, Shuangxi Li, Yingjie Chen, Jian Liu

**Affiliations:** 1 College of Hydraulic and Civil Engineering, Xinjiang Agricultural University, Urumqi 830052, China; an001026@163.com (S.A.); xjlsx123@126.com (S.L.); chenyj2021@xjau.edu.cn (Y.C.); xjliujian117@163.com (J.L.); 2 Xinjiang Key Laboratory of Hydraulic Engineering Security and Water Disasters Prevention, Urumqi 830052, China

**Keywords:** calcium leaching, Portland cement, low-heat cement, C_2_S content, moderate-heat cement

## Abstract

Hydraulic structures are frequently subjected to soft-water or acidic environments, necessitating serious consideration of the long-term effects of calcium leaching on the durability of concrete structures. Three types of common Portland cement (ordinary Portland cement, moderate-heat cement, and low-heat cement) paste samples widely applied to hydraulic concrete were immersed in a 6 mol/L NH_4_Cl solution to simulate accelerated calcium leaching behavior. The mass loss, porosity, leaching depth, compressive strength, and Ca/Si ratio of the three types of pastes were measured at different immersion stages (0, 14, 28, 56, 91, 140, and 180 days). The Vickers hardness index was employed to compare cement samples subjected to erosion for 30 and 180 days. The microstructure and composition of the mineralogical phases of the leached samples were also determined by X-ray diffraction, thermogravimetric analysis, and scanning electronic microscopy. Accordingly, the time-varying behavior and deterioration mechanism of the different cements subjected to leaching were contrastively revealed. The results showed that the calcium leaching resistance of the low-heat cement was the best, followed by the moderate-heat cement and ordinary Portland cement, proving that the content and structure of Ca(OH)_2_ and C-S-H gels were closely related to the leaching performance of the cement. The less Ca(OH)_2_ and more aggregated C-H-S gels produced by C_2_S led to better calcium leaching resistance in the cement. Therefore, the leaching performance of Portland cement could be effectively improved by reducing the content of C_3_S and increasing the content of C_2_S, and the dissolution rate of calcium ions under leaching could be reduced by controlling the low initial calcium content in cementitious materials. This paper offers theoretical guidance for mitigating the long-term effects of calcium leaching on hydraulic concrete structures by conducting a comprehensive comparative analysis of the damage behavior and deterioration mechanisms of various types of Portland cement under identical erosion conditions.

## 1. Introduction

The calcium leaching of hydraulic concrete consists of a process in which cement-based materials are exposed to soft water for long periods of time. Consequently, the equilibrium state between solid calcium in the hydration products and calcium ions in the pore solution will be destroyed due to the concentration difference, resulting in a diffusion loss of the calcium phase in the pore solution and the decalcification dissolution of hydration products, leading to concrete failure [1]. The content of calcium hydroxide (Ca(OH)_2_) and hydrated calcium silicate (C-S-H) gels produced by the hydration of the tricalcium silicate (C_3_S) and dicalcium silicate (C_2_S) of the cement serves as the main factor affecting damage caused by calcium leaching from cementitious materials [2,3]. Currently, scholars have extensively studied the erosion performance and hydration characteristics of ordinary Portland cement (OPC), moderate-heat Portland cement (MHC), and low-heat Portland cement (LHC), with a primary focus on their erosion degradation behavior, mechanical properties, and erosion mechanisms [4,5,6]. For instance, lv et al. [7] found that the leaching resistance coefficient of low-heat cement was higher than that of ordinary Portland cement, and the compressive strength and flexural strength of low-heat cement samples were higher than those of ordinary Portland cement at the same leaching age. Following a 29Si NMR test, Wang et al. [8,9,10] found that the porosity of low-heat cement under complete hydration was significantly lower than that of ordinary Portland cement, and the average molecular chain length of C-S-H gel in the low-heat cement paste was longer than that in the ordinary Portland cement. Wang et al. [11] compared the basic physical properties of low-heat cement and ordinary cement, revealing that the large amount of C-S-H gel produced through the hydration of the low-heat cement dominated by C_2_S was the primary factor leading to a high-strength dense cement-stone structure.

The differences in the contents of calcium silicate (C_3_S) and dicalcium silicate (C_2_S) were the primary factors contributing to the distinct hydration behaviors of the three cement types, ultimately resulting in variations in their calcium leaching performance. [12,13]. Most studies on different types of Portland cement primarily investigate their chemical properties, crack behavior, and strength development [14,15]. Conversely, comprehensive comparative studies on the damage mechanisms of ordinary Portland cement, moderate-heat cement, and low-heat cement under identical erosion conditions, particularly from the perspective of mineral composition, remain relatively scarce [16]. The advantages of the high C_2_S content and low C_3_S content of low-heat cement compared to moderate-heat cement and ordinary cement involve reduced amounts of the hydration product Ca(OH)_2_ and more C-S-H gel, with the hydration equations shown in Equations (1) and (2) [17]. It is generally believed that the leaching resistance of cementitious materials can be effectively improved by reducing the C_3_S content and increasing the C_2_S content. By comparing the mechanical performance of Portland cement with different compositions in 5% sodium sulfate solution, Cao et al. [18] found that cement specimens with low C_3_S and tricalcium aluminate (C_3_A) concentrations exhibited good leaching resistance. Wang et al. [19] found that the early hydration rate of C_2_S was very slow; however, this process could be conducive to the generation of a physically well-structured C-S-H gel. Thus, the internal pore size and the distribution of hydration products in low-heat cement were more homogeneous than those in ordinary cement. He et al. [20] showed that the average chain length and degree of polymerization of hydrated calcium silicate after the full hydration of low-heat cement were higher than those of ordinary Portland cement, which could reduce the decalcification of hydrated calcium silicate due to the exhaustion of Ca(OH)_2_ depletion during the leaching process.

(1) $${2C}_{3}S+11H\to C_{3}S_{2}H_{8}+3\mathrm{CH}$$

(2) $$2C_{2}S+9H\to C_{3}S_{2}H_{8}+CH$$

To simulate long-term calcium leaching within a short period and predict the long-term performance of cementitious materials in low-concentration environments, this study employed a 6 mol/L NH_4_Cl solution to accelerate the erosion of ordinary Portland cement, moderate-heat cement, and low-heat cement samples. Macroscopic indicators, including the mass loss rate, porosity, leaching depth, compressive strength, Vickers hardness, and Ca/Si ratios, were measured for the three types of cement samples. The microstructural morphology and phase composition of the cement samples before and after calcium leaching were analyzed using scanning electron microscopy (SEM), X-ray diffraction (XRD), and thermogravimetric analysis (TGA) to understand the erosion mechanisms involved. By analyzing the calcium leaching characteristics of three types of silicate cement and incorporating microscopic testing, this study examined how variations in mineral compositions and hydration products influenced the calcium leaching damage mechanism of the cement matrix. This comparative analysis provided theoretical guidance for optimizing cement material applications in soft-water or acidic environments. Among the tested cements, the low-heat cement exhibited superior anticalcium leaching performance, underscoring its potential as a sustainable and high-performance material.

## 2. Materials and Methods

### 2.1. Materials and Sample Preparation

Low-heat Portland cement, moderate-heat Portland cement (GB/T 2000-2007 [21], namely ASTM C150 (2018) [22] type IV Portland cement), and P.O 42.5 ordinary Portland cement (GB 175–2023 [23], namely ASTM C150 (2018) type I Portland cement) were used in this study to explore the impact of compositions on their calcium leaching performance. Chemical and mineral compositions, as well as the physical–mechanical properties of the three types of Portland cement, were supplied by Sichuan Jiahua Cement Plant, China. Detailed values are presented in Table 1 and Table 2.

According to the commonly used proportioning scheme for hydraulic concrete, three types of cement paste specimens were designed for Portland cement, denoted as OPC, MHC, and LHC. Cylindrical specimens with 50 mm diameters and 100 mm heights were cast and stored in a standard curing environment (20 °C and 98% relative humidity) for 24 h before demolding. Subsequently, all samples were cured in saturated limewater (Westlong Science & Technology Co., Ltd., Shantou, Guangdong Province, China)at 20 °C until reaching an age of 90 days, followed by accelerated leaching tests in a NH_4_Cl (Westlong Science & Technology Co., Ltd., Shantou, Guangdong Province, China) solution.

### 2.2. Sample and Exposure Conditions

**Figure 1:**
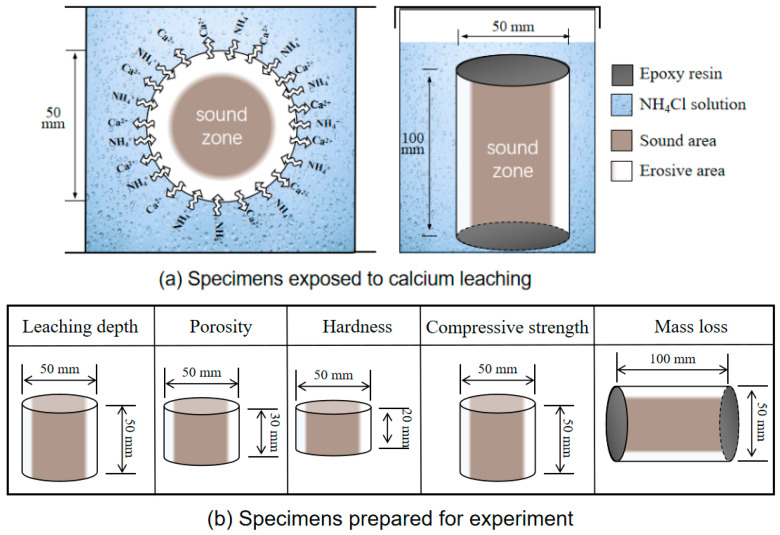
Schematic diagrams showing the exposure conditions and testing.

Prior to the immersion test, epoxy resin (Evergrande New Material Technology Co., Ltd., Huizhou, Guangdong Province, China) was uniformly applied to both ends of the specimens to ensure that the calcium leaching process proceeded in the diameter direction. Then, the specimens were transferred to several sealed boxes filled with 6 mol/L of the NH_4_Cl solution for the full immersion test, as shown in Figure 1a, and all sealed boxes were kept at a constant temperature of 20 °C. During the test, the NH_4_Cl solution was replaced every 30 days to ensure that the concentration of the test solution was stable.

To test the indicators at different immersion stages (0, 14, 28, 56, 91, 140, and 180 days), prior to measuring the test indicators, the samples were sectioned using an HQP-150 concrete core slicing machine (China Construction Instrument and Equipment Co., Ltd., Cangzhou, China). The specimens were cut into small specimens with a diameter of 50 mm and different heights. The porosity, compressive strength, leaching depth, Vickers hardness, and Ca/Si ratio were determined, as shown in Figure 1b. In addition, an electronic balance (0–1 kg range, 0.01 g accuracy) manufactured by Shanghai Puchun Metrology Instruments Company (Shanghai, China) was utilized throughout the experiment. Specific intact cylinders were used throughout the test to determine the mass loss under different leaching ages. Three samples were prepared for each test, and the average value of three data points was used as the result.

### 2.3. Methods

#### 2.3.1. Mass Change and Porosity Tests

Mass loss and porosity serve as important indicators in evaluating the durability of cement paste [24]. The relative mass loss (*W*) and porosity (*P*) of the samples over a certain exposure duration were calculated using Equations (3) and (4):

(3) $$W=\frac{M_{0}-M_{t}}{M_{0}}$$

(4) $$P=\frac{m_{t}-m_{0}}{V}$$

where *M*_0_ and *M_t_* are the weights of the same cylinder specimens before and after calcium leaching, which were air-dried to a constant value in the laboratory before measurement, in g; *m_t_* is the saturated surface dry mass of the subsample (see Figure 1b) in g; *m*_0_ is the mass of this cylinder after drying at 60 °C for 24 h in g; and *V* is the sample volume measured using the drainage method in cm^3^.

#### 2.3.2. Leaching Depth Test

Leaching depth serves as an important indicator for detecting the speed and extent of the calcium leaching process, providing a way to determine micro-leaching behavior from a macro-phenomenon [25]. Cement specimens at the specified immersion stage were removed and split along the diameter using a cutting machine. The residual cement paste on the specimen’s cross-section was rinsed with clean water, and excess surface moisture was dried using absorbent paper. The specimen was then sprayed with 1% alcohol phenolphthalein reagent (Shentai Chemical Reagent Co., Ltd., Tianjin, China), and the distance from the cylinder edge to the discoloration demarcation line was measured as the leaching depth using vernier calipers when the leached specimens appeared obviously colorless with pink demarcation lines [26].

#### 2.3.3. Compressive Strength Test

Compressive strength is considered a fundamental and important mechanical property, as well as a direct index used to quantify the deterioration of specimens [27]. After the cement samples reached the designated calcium leaching stage (0, 14, 28, 56, 91, 140 and 180 days), compressive strength experiments were performed with an electronic universal testing machine at a path-controlled speed of 0.1 mm/min. The strength loss rate ($\Delta \sigma_{t}$) was calculated according to Equations (5) and (6):

(5) $$\sigma_{t}=\frac{F_{t}}{A}$$

(6) $$\Delta \sigma_{t}=\frac{\sigma_{0}-\sigma_{t}}{\sigma_{0}}\times 100\%$$

where *F_t_* is the failure load in kN; *A* is the cross-sectional area of the cylindrical specimen in m^2^; $\sigma_{t}$ is the strength of damage of the sample exposed to NH_4_Cl solution for *t* days in MPa; and $\sigma_{0}$ is the initial compressive strength in MPa.

#### 2.3.4. Vickers Hardness Test

Hardness testing serves as an effective approach for locally and immediately estimating microstructural changes in materials [26]. The Vickers hardness at different leaching depths was measured and combined with the compressive strength loss rate to determine the mechanical property degradation characteristics of cement-based materials under calcium leaching. Prior to testing, all tested sections of the cut subsamples (Figure 1b) were manually polished using 400-, 800-, and 1000-grit sandpapers (Gerde Industrial Technology Co., Ltd., Shanghai, China) with water as a lubricant and then dried in the laboratory for 24 h. The hardness was measured every 0.5 mm from the outer layer of the cement pastes to the interior along the diffusion direction, and the resulting values consisted of the mean values of eight testing points with the same radius. The measurements were carried with a micro-Vickers hardness tester with 0.9807 N of force applied to the surface for 15 s.

#### 2.3.5. Calcium–Silicon Ratio Test

To quantify the changes in the hydration products of the three types of cement pastes with different degrees of leaching, the cylindrical specimens were cut into thin disks with a thickness of 1.5 mm, the calcium ion content of the solution was tested by using the EDTA complexometric titration method, and the Ca/Si of the entire specimen was accordingly determined [28]. To calculate the calcium content of the leached specimens and to obtain the Ca/Si ratio of the disks in the leaching solution, we subtracted the calcium content in the leaching solution from the calcium content of the initial disk.

#### 2.3.6. Mineralogical, Morphological, Thermal, and Microstructural Analyses

After leaching in NH_4_Cl solution for 180 days, several typical samples were collected from the outer layer of the damaged specimens by cutting and artificially breaking off the specimens. We then conducted X-ray diffraction (XRD), thermogravimetric analysis (TGA), and scanning electronic microscopy (SEM) assessments. The samples at the designated erosion stage were ground into powder with a particle size smaller than 80 μm and then dried in a glass vessel with a desiccant at room temperature until the XRD and TGA measurements were performed. The XRD analysis was performed using a Rigaku SmartLab SE (Rigaku Corporation, Tokyo, Japan) diffractometer with a 40 kV operating voltage, a scanning speed of 10°/min, and a scanning range of 10–50° to assess changes in the specimens’ crystal compositions during the erosion process. The thermogravimetric analysis (TGA) was performed using a NETZSCH STA 449F3 synchronous thermal analyzer (NETZSCH-Gerätebau GmbH, Selb, Germany) under a nitrogen atmosphere, with a temperature range of 30–600 °C and a heating rate of 10 °C/min. Moreover, the obtained fracture surfaces of the flake samples maintained their original micro-morphology, and they were subsequently coated with a 5 nm thick gold film and observed with a TESCAN MIRA LMS-type scanning electron microscope (TESCAN ORSAY HOLDING, a.s., Brno, Czech Republic).

## 3. Results and Discussion

### 3.1. Mass Loss Rate

**Figure 2:**
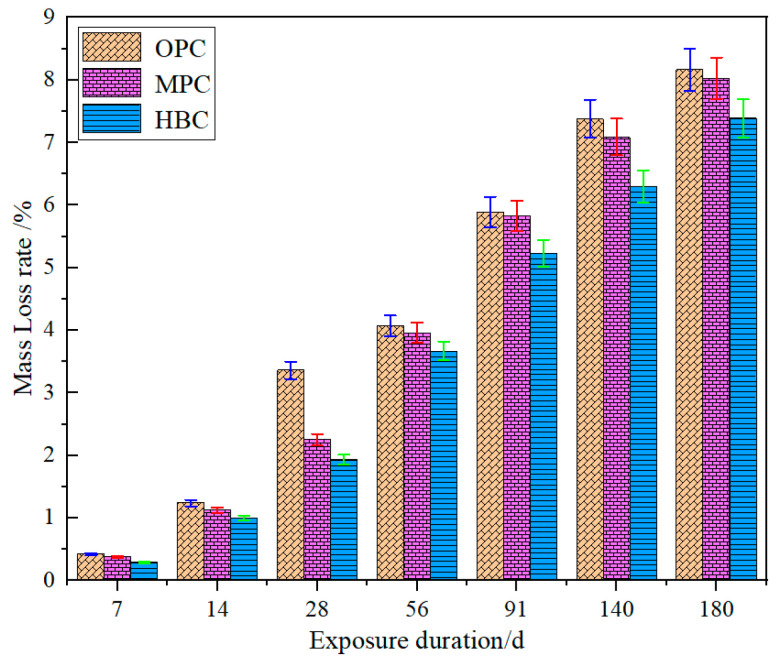
Mass loss rates of specimens exposed to NH_4_Cl solution.

Figure 2 shows the change in the mass loss rate of the three types of Portland cement specimens in the 6 mol/L NH_4_Cl solution. The results show that the mass loss rates of the OPC, MHC, and LHC specimens subjected to accelerated leaching with the NH_4_Cl solution all increased with increasing immersion time. After 180 days of leaching, the mass loss rates of the OPC specimen was greater than that of the MHC and LHC, with values of 8.17%, 8.03%, and 7.39%. For the OPC specimen, the mass loss rate was higher than that of the other two specimens during the 180-day leaching period. Therefore, it could be assumed that under the same leaching conditions, the resistance to calcium leaching of the ordinary cement was weaker than that of the moderate-heat and low-heat cements and that the latter, by having a decreased content of C_3_S and increased content of C_2_S, effectively reduced the generation of hydration products of Ca(OH)_2_ and reduced the loss of the calcium phase from its cement specimens in the process of calcium leaching.

### 3.2. Porosity

**Figure 3:**
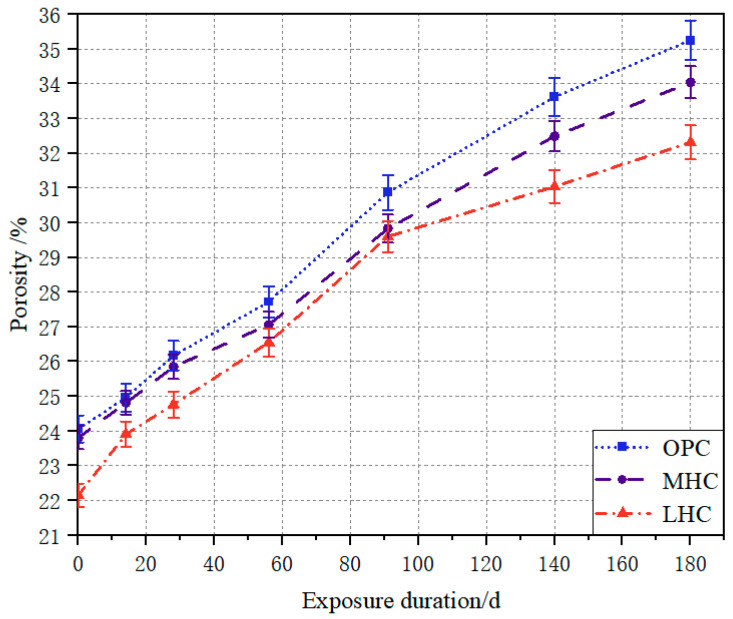
Porosity of the specimens exposed to NH_4_Cl solution.

The porosity development of the OPC, MHC, and LHC specimens subjected to the NH_4_Cl solution is shown in Figure 3, where the continuous depletion of the calcium phase in the hydration products was the main reason for the increase in porosity. The porosity of the cement specimens eroded over 180 days followed the order of OPC > MHC > LHC, from large to small. In addition, the porosity of the ordinary cement specimen showed the highest increase of 11.19% throughout the leaching process, followed by 10.23% for the moderate-heat cement specimen and the smallest increase of 10.16% for the low-heat cement specimen. The slower growth in porosity of the MHC and LHC specimens compared to the OPC specimen under accelerated leaching with the 6 mol/L NH_4_Cl solution was consistent with the change in the mass loss of all three cement types. This could be attributed to the effect of the hydrated calcium silicate gel produced through silicate cement hydration on the structural densification aspect, while the low-heat cement had the highest C_2_S content and formed the greatest amount of C-S-H gel and the pore structure of its cement specimens was denser [29,30].

### 3.3. Leaching Depth

**Figure 4:**
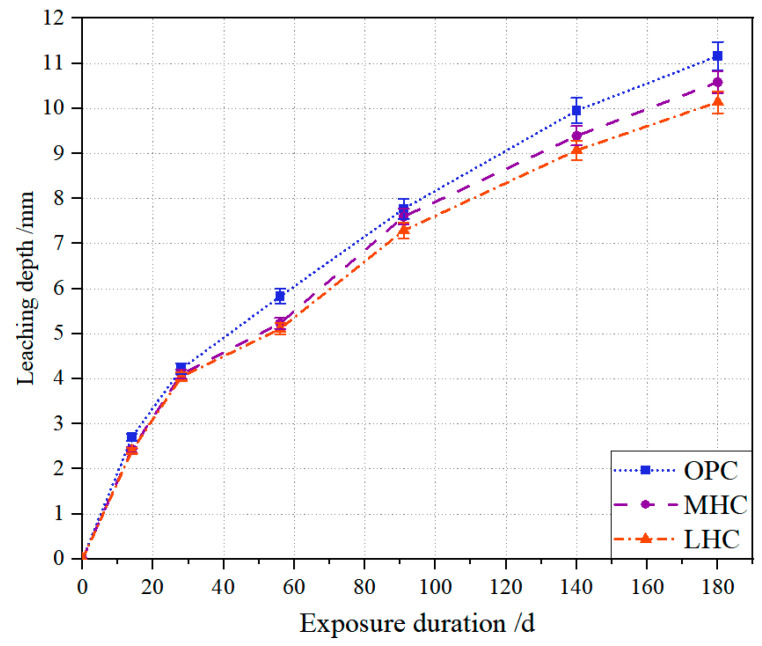
Leching depths of specimens exposed to NH_4_Cl solution.

Calcium leaching behavior is a process that occurs from the outside to the inside, and the leaching depths of the eroded specimens subjected to the NH_4_Cl solution are shown in Figure 4. The leaching depths of the three types of cement pastes increased with the immersion time, and the leaching depths of the OPC specimens at each leaching stage were the largest, followed by those of the MHC specimens, and the leaching depths of the LHC specimens were the lowest. The reason for this was consistent with the change in porosity, where the higher C_2_S content of the low-heat cement specimens generated a large amount of C-S-H gel and a smaller amount of Ca(OH)_2_, which allowed the polymerization and hinge system of the LHC paste to provide a better combination, making it difficult for NH_4_^+^ to enter the interior of the specimen.

### 3.4. Compressive Strength

**Figure 5:**
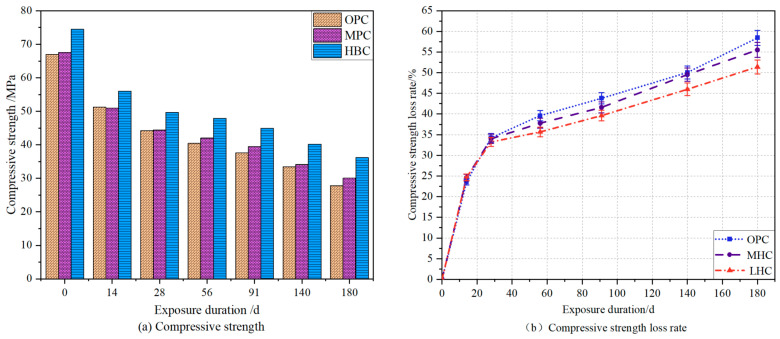
Compressive strength loss rate of the specimens exposed to NH_4_Cl solution.

The results for the compressive strength and loss rate of the three types of Portland cement eroded by the NH_4_Cl solution (0, 14, 28, 56, 91, 140, and 180 days) are shown in Figure 5. These results demonstrate that the compressive strength loss rate of all the cement specimens showed a significant decrease in the first period, followed by a slow decrease. Specifically, OPC, MHC, and LHC exhibited a significant decrease in compressive strength loss rate after 30 days of accelerated leaching, mainly due to the consumption of a large amount of solid-phase Ca(OH)_2_, and subsequently, when the solid-phase Ca(OH)_2_ was almost completely dissolved, a small amount of calcium in the C-S-H gels started to decompose and replenish the local chemical equilibrium, until finally, only SiO_2_ gel remained [31]. The loss rates of compressive strength for the OPC, MHC, and LHC were 58.43%, 55.51%, and 51.4% following the 180-day leaching process, indicating that the more calcium hydroxide produced by the hydration of C_3_S and C_2_S in the cementitious materials, the more serious the deterioration damage to the specimens under calcium leaching. Calcium hydroxide is the component with the highest hardness in cement hydration products, which may be an important reason for the decrease in mechanical properties of cement-based materials [32,33].

### 3.5. Vickers Hardness

**Figure 6:**
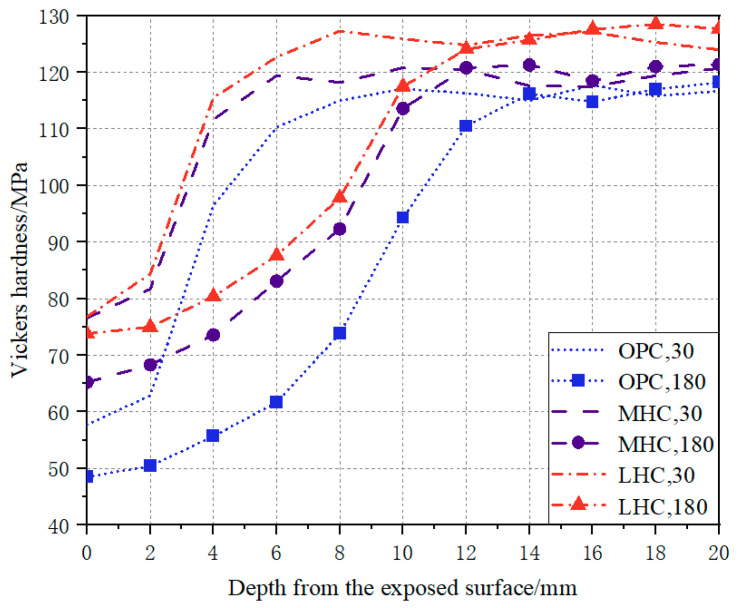
Vickers hardness of the different cement pastes under calcium leaching.

Figure 6 depicts the trend in hardness with depth for the three types of cement specimens subjected to the NH_4_Cl solution for 30 and 180 days. The results show that the Vickers hardness gradually increased from the outer to the inner layers of the specimen and then tended toward a stable value. The Vickers hardness of the LHC specimen in the intact zone was greater than that of the MHC and OPC specimens, which proved that the densification and strength of the hydration products of the low-heat cement with a high C_2_S content were greater than those of the hydration products of the moderate-heat cement and ordinary cement under the same conditions. In addition, the hardness of the three types of specimens in the leaching zone was greatest for the low-heat cement, followed by the moderate-heat cement and then the ordinary cement. The OPC specimens demonstrated the highest loss of the calcium phase and the most serious damage to the internal structure after leaching. Combined with the index of the loss of compressive strength, the mechanical properties also showed that the low-heat cement demonstrated better resistance to calcium leaching.

### 3.6. Ca/Si Variations with Mass Loss

**Figure 7:**
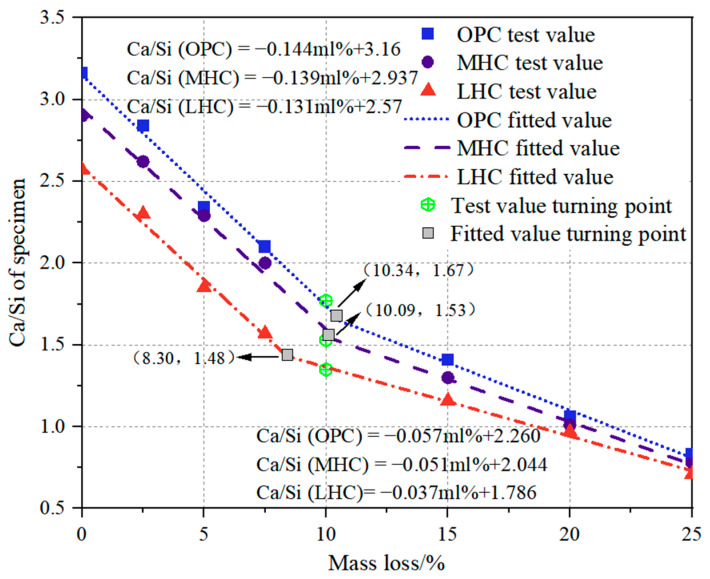
Ca/Si ratio versus mass loss of cement specimens under calcium leaching.

A relationship between the Ca/Si ratio and mass loss was established, as shown in Figure 7. The figure reveals that the Ca/Si ratios of the ordinary Portland cement (OPC), moderate-heat cement (MHC), and low-heat cement (LHC) specimens followed a two-stage linear relationship with mass loss. The slopes of the fitting lines and the inflection points varied among the three types of cement. The Ca/Si ratio decreased as mass loss increased, with a significantly steeper decline in the first stage than in the second stage. The rates of decline in the Ca/Si ratio for the OPC specimen was 0.144 and 0.057 in the first and second stages, respectively, exceeding the rates of 0.139 and 0.051 for the MPC specimen and 0.131 and 0.037 for the HBC specimen. In addition, at the inflection point of the two phases, the OPC corresponded to a transverse mass loss rate of 10.34%, at which point the Ca/Si value was 1.67, while the MHC and LHC corresponded to a decrease in the transverse mass loss rate and Ca/Si ratio at the cutoff point, with values of 10.09% and 1.53 and 8.30% and 1.48.

We inferred that the C_3_S content was higher in the first stage of the OPC and MHC, with more calcium hydroxide produced by hydration, and that Ca(OH)_2_ could easily decompose during the calcium leaching process. Thus, the mass loss and calcium loss were greater than those of the low-heat cement. When Ca(OH)_2_ was almost completely dissolved, following the second phase, the calcium phase in the C-S-H gel started to decompose to maintain the local chemical equilibrium, until finally, only the SiO_2_ gel remained. Due to its high content of C_2_S, the LHC produced less calcium hydroxide from hydration and more hydrated calcium silicate gels, resulting in a lower rate of calcium leaching. Incidentally, this was reflected in the relationship between mass loss and the Ca/Si ratio, as shown in Figure 7, which was also consistent with the law studied by Chen et al. [34].

### 3.7. XRD

**Figure 8:**
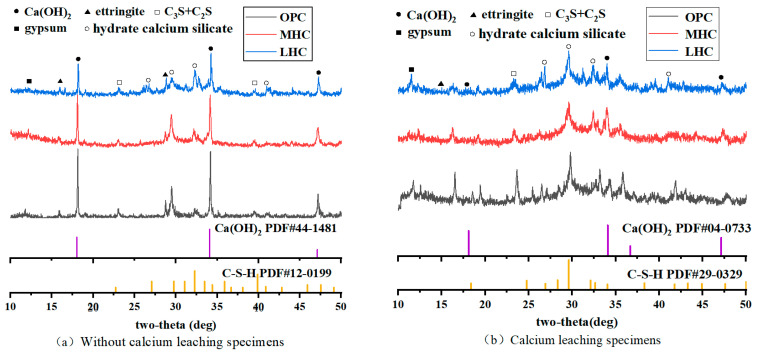
XRD patterns of the cement pastes (**a**) before calcium leaching and (**b**) after being exposed for 180 days.

The compositions of the mineralogical phases of the three types of cement before calcium leaching are shown in Figure 8a. Strong diffraction peaks are shown at 18.1°, 34.2°, and 47.5°, which were observed in the range of 10–50° before the leaching effect of the cement pastes, corresponding to the diffraction peaks of calcium hydroxide [35]. The Ca(OH)_2_ peaks of the three types of cement demonstrated the highest trend for the OPC, followed by the MHC, and the lowest trend for the LHC, which was mainly due to the fact that the C_3_S content of the LHC was the lowest among the three cements and the calcium hydroxide content in the LHC paste was significantly lower than that in the OPC and MHC pastes. The diffraction peaks at 16.0° and 28.9° correspond to those of ettringite (AFt) [36]. The mineralogical phase compositions of the three types of Portland cement after 90 days of calcium leaching are shown in Figure 8b. In the XRD plots of 10–50°, the wider dispersion peaks originated from the amorphous phases of C_3_S and C_2_S [37], which slightly decreased with the onset of the calcium leaching process, indicating that their amorphous phases were consumed during the calcium leaching process. A comparison with the Ca(OH)_2_ diffraction peaks before calcium leaching clearly showed that the calcium hydroxide consumption of the OPC was the greatest after leaching, followed by that of the MHC, and the lowest was observed for the LHC, and these trends were consistent with the Ca/Si ratio.

### 3.8. TGA

**Figure 9:**
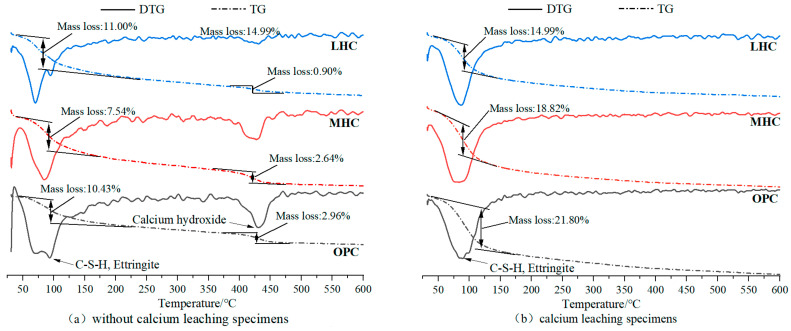
TG-DTG curves of the cement pastes (**a**) before calcium leaching and (**b**) after being exposed for 180 days.

Figure 9 shows the TG-DTG curves of the OPC, MHC, and LHC specimens before and after calcium leaching. As shown in Figure 9, the heat absorption peak in the range of 80–100 °C is mainly related to substances such as the C-S-H gel and AFt, indicating that the LHC specimens before calcium leaching had the highest content of hydrated calcium silicate and calcite from hydration, followed by the MHC and OPC. Based on the porosity and compressive strength indicators before dissolution, along with the high-density characteristics of the calcium silicate hydrate microstructure [38], it was concluded that the formation of abundant calcium silicate hydrate effectively filled the pores in the cement matrix, disrupted pore connectivity, and enhanced the density of the LHC specimens. In addition, the heat absorption peak in the range of 400–480 °C was caused by the dehydration of calcium hydroxide [39], with the Ca(OH)_2_ content following the order of OPC > MHC > LHC, from highest to lowest. With accelerated leaching caused by the NH_4_Cl solution, we found that Ca(OH)_2_ in the range of 400–480 °C was almost completely consumed, while the peaks of the C-S-H gel and AFt in the range of 80–100 °C in the cementitious material showed less change. This indicated that the cementitious material system lost a large amount of Ca^2+^ during the leaching process, and the Ca^2+^ loss was mainly related to the depletion of calcium hydroxide, indicating that Ca(OH)_2_ was the first to be consumed under calcium leaching, followed by calcium phases such as the C-S-H gel [40], which was consistent with the conclusion that the calcium leaching process was divided into two phases, namely the decomposition of calcium hydroxide and that of hydrated calcium silicate, as indicated by the Ca/Si test.

### 3.9. SEM

**Figure 10:**
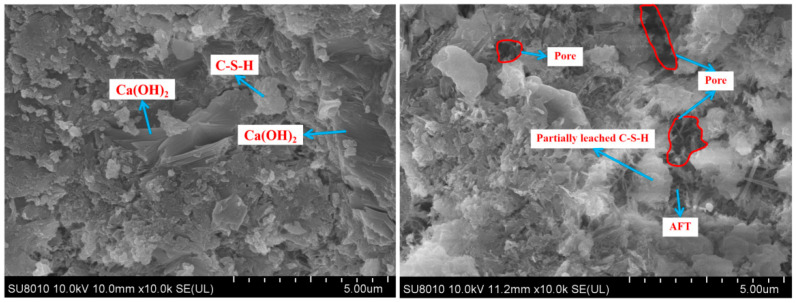
Morphology of the OPC specimens exposed to NH_4_Cl leaching.

**Figure 11:**
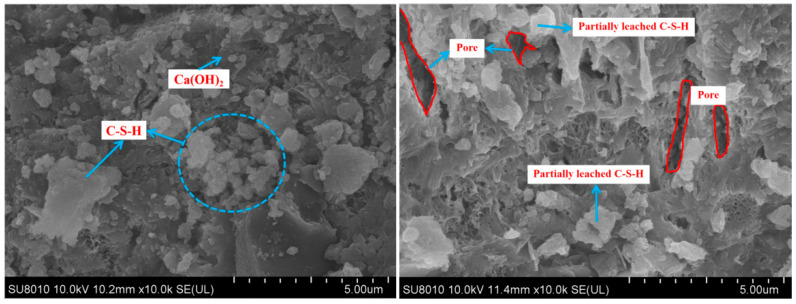
Morphology of the MHC specimens exposed to NH_4_Cl leaching.

**Figure 12:**
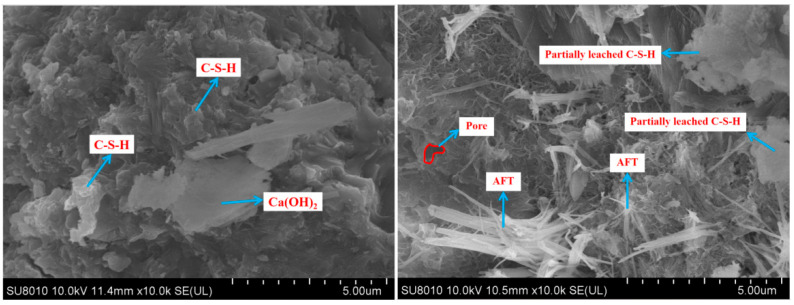
Morphology of the LHC specimens exposed to NH_4_Cl leaching.

The micro-morphologies of the three types of Portland cement pastes before and after calcium leaching were compared using SEM at 2000×, as shown in Figure 10, Figure 11 and Figure 12. The microstructural images of the cement samples before dissolution revealed that, after complete hydration, their hydration products were relatively intact. The OPC sample predominantly consisted of plate-like calcium hydroxide, the moderate-heat cement exhibited denser amounts of C-S-H gel, and the low-heat cement featured high-density C-S-H gel with a more uniform and compact microstructure. This trend was similar to the mechanical properties of the LHC, which demonstrated improved durability compared to the MHC and OPC. After 90 days of calcium leaching, we observed that on the surface of the three types of specimens, the hydration products were significantly reduced, the internal pores significantly increased, and the OPC and MHC specimens were the most seriously damaged, with a significant increase in black pores. The reason for this phenomenon was that the cement specimens under leaching consumed a large amount of solid-phase calcium, such as Ca(OH)_2_ and C-S-H gel, and the cement specimen pores continued to increase, which further increased calcium leaching. Furthermore, in this process, the tricalcium aluminate (C_3_A) inside the concrete specimen generated flaky Friedel’s salt with CaCl_2_ and water. However, the C_3_A content of the OPC was the highest, and this flaky Friedel’s salt possibly had a negative effect on the stability of the internal structure of the specimen, causing expansion damage to the cemented specimen, which was possibly one of the factors that led to the weaker resistance to solvency of the OPC than the MHC and LHC in the NH_4_Cl solution.

## 4. Conclusions

The macro-properties and micro-indices of the ordinary Portland cement, moderate-heat cement, and low-heat cement pastes exposed to the 6 mol/L NH_4_Cl solution were contrastively determined to reveal their calcium leaching behavior and deterioration mechanisms. The following conclusions were drawn based on this work.

(1) The content of Ca(OH)2 in the low-heat cement paste cured for 90 days was significantly lower than that of the ordinary Portland cement and moderate-heat cement, with the largest generation of C-S-H gel being observed in the low-heat cement. Therefore, the internal pore structure of the low-heat cement was the most dense out of the three types of Portland cement after complete hydration.
(2) The macroscopic performance deterioration of the low-heat cement specimens under accelerated leaching was consistent with that of the ordinary Portland cement and moderate-heat cement specimens, and its comprehensive resistance to calcium leaching was the highest, followed by the moderate-heat cement and the ordinary Portland cement.
(3) The large amount of C-S-H gel produced by the hydration of C2S and C3S in the low-heat cement could have improved its calcium leaching resistance, and it could be considered that the leaching resistance of Portland cement could be effectively promoted by decreasing its content of C3S and appropriately increasing its C2S content.
(4) The Ca/Si ratio of the low-heat cement and its leaching rates in both the Ca(OH)2 dissolution stage and C-S-H gel decomposition stage were the lowest out of the three cement types. This was due to the low initial Ca(OH)2 content in the LHC, leading to a lower diffusive dissolution rate of calcium ions, and the C-S-H gel in the LHC pastes exhibited a higher degree of polymerization, which made it relatively more difficult to decompose.

## Figures and Tables

**Table 1:** Chemical composition and mineral composition of cement (wt.%).

Sample Codes	Chemical Composition								Mineral Composition			
	CaO	SiO_2_	Al_2_O_3_	Fe_2_O_3_	MgO	SO_3_	* R_2_O	LOI	C_3_S	C_2_S	C_3_A	C_4_AF
LHC	58.74	22.82	3.55	4.28	4.99	2.43	0.39	1.36	28.7	43.9	2.1	13.0
MHC	60.27	22.18	4.01	4.49	5.07	2.04	0.46	1.06	37.5	35.5	3.0	13.6
OPC	62.83	20.50	5.61	3.84	1.70	3.07	1.31	1.59	48.0	22.9	8.4	11.7

Note: * R_2_O = Na_2_O + 0.658K_2_O; C_4_AF: tetracalcium aluminoferrite.

**Table 2:** Physical and mechanical properties of cement.

Sample Codes	Density /(g·m^3^)	SSA /(m^2^·kg^−1^)	Setting Time/min		Soundness	CS/MPa		
			Initial	Final		7d	28d	90d
LHC	3.21	332	233	343	Pass	25.4	53.6	69.8
MHC	3.22	325	227	332	Pass	30.7	51.4	65.0
OPC	3.22	356	125	277	Pass	41.5	57.1	65.3

## Data Availability

The original contributions presented in this study are included in the article. Further inquiries can be directed to the corresponding author(s).
